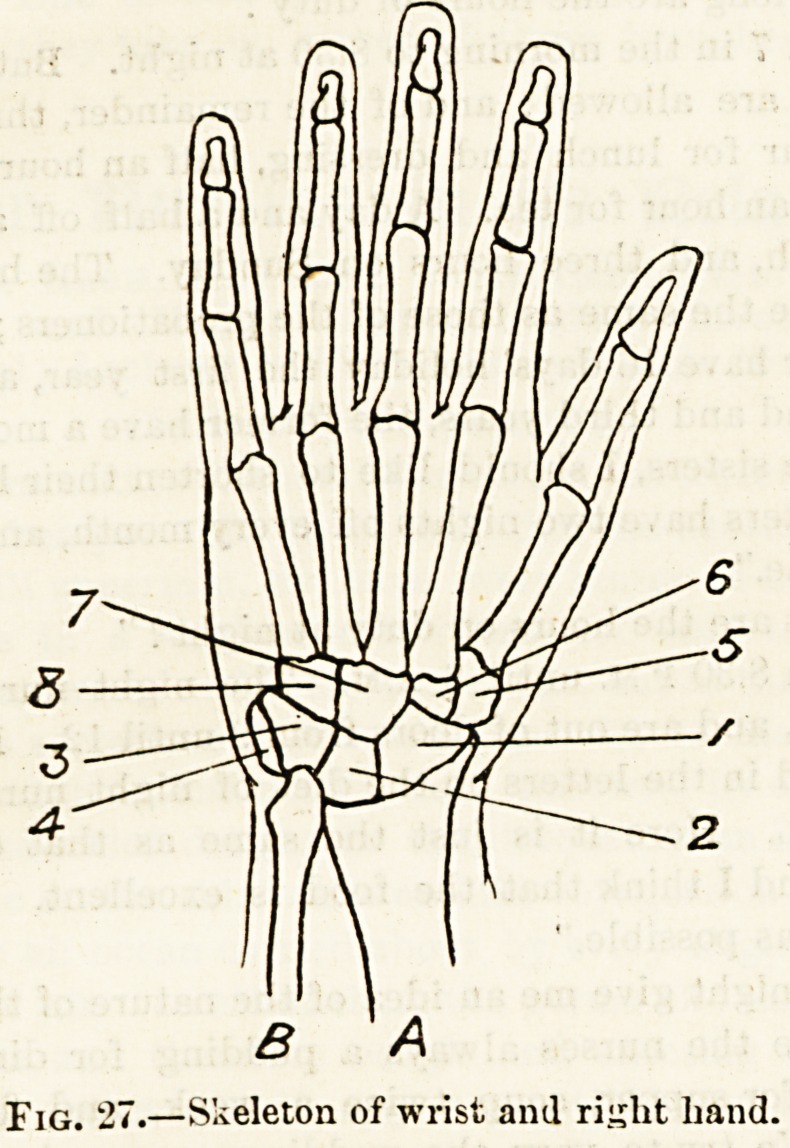# The Hospital. Nursing Section

**Published:** 1902-02-22

**Authors:** 


### The Hospital.

### Nursing Section.

Contributions for this Section of "The Hospital" should be addressed to the Editor, "The Hospital" Nursing Section, 28 & 29 Southampton Street, Strand, London, W.C.

No. 804.—Vol. XXXI. *SATURDAY, FEBRUARY 22, 1902.*

### Notes on News from the Nursing World.

### QUEEN ALEXANDRA'S NAVAL NURSING SERVICE.

Her Majesty, Queen Alexandra, has consented to become President of the Naval Nursing Service, which will therefore henceforth bear her name, in the same manner as the Military Nursing Service. That the Queen takes just as keen an interest in the welfare of the Navy, which her son the Prince of Wales selected as his profession and followed with enthusiasm, as in that of the Army, has never been doubted. But her gracious act in thus personally identifying herself with the service will be warmly appreciated by all its branches, and especially by those who have the privilege of belonging to the nursing staff.

### THE WAR NURSES.

The following nursing sisters arrived at Southampton from South Africa on board the *Roslin Castle* on Tuesday last week:—C. Harris, requires one month's leave; A. M. Harrison, requires one month's leave and returns to South Africa; E. C. O. Legett, time-expired; K. E. Nisbet, time-expired. All the above are members of the A.N.S.R. Nursing Sister S. A. C. Jones was invalided home, but returns after six months' leave. The following disembarked from the *Plassy* on Wednesday, the 12th:—Acting Superintendent G. E. Saunders, A.N.S., requires two months' leave and returns to South Africa; M. A. Bindloss, A.N.S.R., requires one month's leave and returns to South Africa; J. Butler, A.N.S.R., requires one month's leave, and possibly an extension, and returns to South Africa; and Civil Nurse J. C. Child, requires six months' leave and returns to South Africa.

### AN ENGLISH NURSE ON THE CONCENTRATION CAMPS.

Miss Brereton, of Guy's Hospital, who was one of the superintending nurses of the Yeomanry hospitals in South Africa, and was appointed by the Secretary for War as a Commissioner to visit and report upon the Concentration Camps, has just returned to England. In the course of the inquiry she visited every Boer camp but one, and upon her arrival at her home in Norfolk, where she was received with enthusiasm by her friends and the villagers, she delivered a little speech, in the course of which she said:—"Whatever mistakes have been made—and mistakes cannot be avoided in experiments, I am sure the best has been done for these people. In the main the Boer people are intensely surprised at the treatment they have received from soldiers they had always been told were brutes, but that now, in their own words, they find 'quite good.'" Miss Brereton's practical knowledge renders this statement valuable.

### NEW SOMERSET HOSPITAL, CAPETOWN.

At the monthly meeting in December of the Committee of the New Somerset Hospital, Capetown, the matron's salary was fixed at £120 per annum, increasing yearly to £150. It was decided to re-advertise the vacancy, and that the Agent-General and Dr. Patsons (for many years house surgeon) should be asked to help in selecting a suitable candidate. In future an assistant matron will be appointed to undertake the housekeeping at a salary of £100 per annum, rising to £120. Improvements have been made with regard to the increased number of night nurses, and stricter rules have been enforced as to the presence of nurses in their wards, and other methods of discipline. A regular system of lectures is being kept up, and the result of the nurses' examination by the Colonial Medical Council is satisfactory. The Committee were pleased to hear the opinion of the resident surgeon that the quality of the nurses ranks high as compared with those engaged in similar institutions in Great Britain.

### PROGRESS AT ST. PANCRAS INFIRMARY.

The interview of our Commissioner with Miss Moir, matron of St. Pancras Infirmary, reported in another part of the paper, shows once more that under the régime of a highly trained matron, and with the co-operation of an enlightened Board of Guardians, the nursing at a workhouse infirmary may be brought up to an excellent level. The sleeping accommodation at St. Pancras Infirmary is not so good as it ought to be, but it will be seen that material progress has been made in this respect, while in others there is little to complain of. We can understand that there is a difficulty about the rations. There should be some elasticity in the system, and a certain measure of discretionary power should be entrusted to a matron in whom the guardians have every confidence.

### DISGRACEFUL NEGLECT AT MILE END.

It has at length been allowed to transpire that of the seven nurses who caught small-pox at Mile End Infirmary, not one had been vaccinated. We, of course, suspected that this was the case, particularly when an inquiry which we made on the subject was ignored, but, until specific information was forthcoming, we did not think it desirable to assume that the authorities of Mile End Infirmary had dared to permit nurses to come into contact with small-pox without being re-vaccinated. The obviously proper precaution is for every new member of the nursing staff of every hospital or infirmary to be vaccinated before she commences her duties. But in this instance there were additional reasons for insisting upon it, and if any nurse connected with Mile End Infirmary loses her life from the disease, the authorities of the Union will be morally responsible for her death.

### LEGAL RESPONSIBILITY FOR CARELESSNESS.

The unfortunate mistake whereby a small-pox patient in the Orsett Isolation Hospital lost his life, is a timely warning to all engaged in nursing work to exercise more than ordinary care with regard to the disposition of medicines and disinfectants. With the jury's expression of sympathy for the nurse we cordially agree, and would not willingly add anything to accentuate the regret which she must feel under the painful circumstances. But we are constrained to point out that, although according to the evidence the patient did not die from the effect of the poison accidentally taken, yet the reported facts are an illustration of a kind of negligence which might have results entailing responsibility of a far different kind. We take the law to be now well settled, that if a person by an act of gross negligence causes death, he is open to a charge of manslaughter, though the punishment may be mitigated, or nominal, if the negligence was no more than inadvertence. At the same time it must not be forgotten that a person in the position of a trained nurse has a duty of extra care placed upon her with regard to the administration of medicines to her patient, which would render it more difficult for her to withstand a charge of criminal negligence than if the act causing the death were that of an unskilled person. For this reason we would draw attention to this case, the report of which might, without a very careful perusal, lead to the inference that carelessness has no legal consequences to be feared. In fact, this is by no means the case; and we hope that the lesson which this fatality conveys may be read, marked, learned, and inwardly digested.

### QUALIFICATION OF NURSES.

Miss Stevenson, a member of the Board of Management of the Royal Edinburgh Infirmary, in an address last week at Manchester on "Women on Hospital Boards," dealt with the question of the qualification of nurses. In the past, she said, the public had been too confiding with regard to the qualifications of nurses sent to them, and had not inquired into their training. This is true; but Miss Stevenson makes the mistake of advocating State registration as a remedy, for it would in practice prove nothing of the kind. She also urges that nurses receiving training should "receive no pay beyond board and lodging, uniform, and other perquisites." We do not think that such a change would be either for the advantage of nurses or for the benefit of the public. The remedy for putting a stop to the practice of adventuresses posing as nurses, and of supplying the public with full and reliable information as to training, is already provided by the annual publication of "Burdett's Official Nursing Directory." If medical men, public bodies, and individuals, would make a point of not engaging a nurse whose adequate training cannot be proved by a reference to the "Official Directory," there would be no complaints about untrained persons palming themselves off as genuine nurses. Miss Stevenson does not seem to attach much importance to the usual hospital certificate; but she does not suggest a substitute. If the school in which a nurse has been trained cannot judge of her fitness, and is not the best authority to issue the certificate of competency, who can testify to these necessary attributes of a nurse?

### GUARDIANS AND DISTRICT ASSOCIATIONS.

Both at Shrewsbury and Crediton the question of the contribution by Guardians to district nursing associations has just been discussed. At Crediton, we regret to say, an attempt to induce the Guardians to reconsider their refusal to subscribe annually to the funds of the Nursing Association was defeated by a majority of one, though Sir John Shelley and the Rev. R. Knight, two of the most influential members of the Board, insisted that it was their duty to render assistance. Mr. Heighway Jones and Dr. Cureton were more successful at Shrewsbury, for when they had advocated a subscription to the funds of the Pontesbury District Nursing Association, and the proposition was put to the Atcham Board of Guardians, it was carried by 14 to 9. The only plausible objection put forward was that if the Pontesbury Association received a contribution, other organisations in the district would also expect one. But even if all the associations, of which there are only four, were given three guineas a year the amount would be small. Moreover, we agree with Dr. Cureton that subscriptions of this character tend rather to relieve than to burden the ratepayers, since it must obviously cost more to nurse the sick poor in the workhouse infirmary than by means of the district nurse in their own homes.

### A DANCE AT A NURSING INSTITUTE.

There has been a nurses' dance at Northampton in the newly-opened Diamond Jubilee Nursing Institute, and the cost was defrayed by a few friends. The guests numbered about 35, and the nurses' room and the dining-room were set apart for dancing. The nurses attached to the Northampton Nursing Institution will not, we are sure, discharge their duties less satisfactorily because they enjoy a little recreation, and if there were no patients in the institute at the time, no exception need be taken to the incident. But otherwise an institute stands on the same footing as a hospital or an infirmary, and we think that dancing would be better elsewhere. The Northampton nurses paid no less than 12,226 visits last year, which is an excellent record, and we are glad to learn that with another £150 to complete the furnishing, and £90 for provision for the storage of Bath chairs and cycles, the beautiful new home will be free of debt.

### HOME OF REST FOR NURSES

The Committee of the Almondsbury Memorial Hospital and Institute, near Bristol, have decided upon a new departure. The building was erected in 1893 by the late Mr. Sholto V. Hare, for the benefit, primarily, of the parishioners of Almondsbury and district. One wing is in use as a cottage hospital, the main block being originally intended for parish rooms, reading room and club rooms. The new schools being now employed for those purposes, the large airy rooms at the institute are vacant, and as there is a very insufficient endowment fund the committee have decided to open a Home of Rest, where hospital nurses, convalescent or otherwise, can be received on very reasonable terms, the proceeds being devoted to the support of the hospital. Six beds will be available. The district is admirable for cycling, and there is ample accommodation for machines. Should the weather prove unfavourable the large parish room can be utilised for ping-pong and other games. The institute being within easy reach of Bristol it should prove a boon to nurses glad of real rest, pure air, lovely scenery and country fare combined, without the restrictions of an ordinary convalescent home. "Breakfast in bed allowed" is an item that will commend itself to tired workers. Colonel and Mrs. Chester-Master, of Knole Park, have kindly thrown open the whole of their extensive grounds to nurses who may avail themselves of the hospitality of the institute, and they will be sure of a warm welcome from the matron who has herself been for two and a half years in charge of the nurses' sick room of a large general hospital.

### GOOD WORK AT BURY.

The fifth annual report of the Bury Branch of Queen Victoria's Jubilee Institute for Nurses reflects credit on the Lancashire town. Work has steadily increased, the number of visits paid during 1901 being 9,485 as against 7,764 in 1900, while the balance at the Bank stands at £76 10s. 6d. The sum of £1,000 has been given in memory of Queen Victoria to the trustees of the Society, to be devoted to the funds for maintaining the nurses in Bury; the Police Sports Committee gave £10; the Co-operative Society the same sum; a dramatic entertainment yielded £35; and the Girls' Grammar School and others have all helped. Grateful patients contributed £12 6s. 6d., instead of the small sum of £4, the total of the year before. The breadwinner of the family has grown to welcome the nurse's visits. In 1900 only 44 men were nursed, in 1901 the number was 101.

### NEWTON ABBOT WORKHOUSE INFIRMARY.

The late superintendent nurse of Newton Abbot Workhouse Infirmary takes away some pleasing mementoes of her three and a half years' labours. The present made on Monday on behalf of the officers was a handsome gold watch, which the medical officer handed to Miss Fisher, who also received a gold bracelet from her grateful patients. She is about to assume the position of superintendent nurse at Whitechapel Infirmary. The Newton Guardians have not yet filled the vacancy. They have advertised but have not attracted many candidates, and did not make a selection from those who sought the position. On the motion of Dr. Ley, however, they took a step forward by deciding to offer a better salary, and the new superintendent will start at £45, rising to a maximum of £50 per annum.

### INCORPORATED SOCIETY OF TRAINED MASSEUSES.

The members of the Incorporated Society of Trained Masseuses held their annual meeting on Friday last, at the Trained Nurses' Club, 12 Buckingham Street, Strand. There was a good attendance, some of the masseuses coming from a considerable distance in order to be present. The meeting was an animated one, and a very general interest was evinced in the business of the evening. The announcement of Sir William Bennett's kind promise of a lecture to the members of the society in March was received with enthusiasm.

### A BUSY SEASON FOR PRIVATE NURSES.

It was stated the other other day in the daily press that the demand for private nurses is so great at the present time that most of the leading nursing homes in West London were unable to meet it. This seems to be true, and the head of the South Kensington Nurses' Co operation informed our representative that she has been "living on the telephone," and that while the nurses on her staff are always fairly busy with surgical cases, the rush of medical cases has proved too great to be met. Last week more than a dozen applications were refused, after communication had been made by telephone with several of the leading institutions, including the Nurses' Co-operation, Brompton Hospital, the Sisters of St. John the Divine, who also had no one at liberty. At other institutions, even as far west as Ealing, the same tale was told, and the secretary of the Auxiliary Nursing Association, 10 Orchard Street, is hard at work too. She assured our representative that if any really good nurses apply for membership, she will be very glad to bring their names forward, and that if the epidemic of influenza lasts it is probable that there will be plenty to do. The private staff of the Middlesex Hospital is also fully employed, and the Chartered Nurses' Society and the Nurses' Co-operation have been applied to for help. But this is always the case in February, and the Matron of the Middlesex does not consider that the present time is one of abnormal stress, taking all things into consideration.

### OFFICIAL RECEPTION AT EDINBURGH CITY HOSPITAL.

On Monday evening, February 10th, the Corporation of Edinburgh held a reception for the nurses of their own hospital and those of Edinburgh generally in the Music Hall and Assembly Rooms. There were over 400 nurses present in uniform, over 60 belonging to the City Hospital. The matrons present were:—Miss Sandford, of the City Hospital; Miss Wade, of the Queen's Nurses; Miss Beveridge, of the Longmore Hospital; Miss Strange, of Chalmers Hospital; Miss Edwards, of the Maternity; Miss Peter, of Craig House; Miss Stewart, of Campie House; the matrons of the poorhouse hospitals, private nursing institutions, and asylums; also Miss Adams, of Ruchill Fever Hospital, Glasgow. The nurses were presented to the senior magistrate by the City Officer, and it was a pretty sight as they walked past, each set headed by their matron. The hospital uniforms were attractive and neat, but some of the private nurses were conspicuous for general untidyness and a want of nurselike appearance. They looked as if they were got up for a fancy ball. Two army sisters were present and their dresses were very charming. The Queen's nurses wore businesslike costumes.

### SHORT ITEMS.

There have been several vacancies for staff nurses at Kimberley Hospital.—Some of the sisters of the Yeomanry Hospital, Elandsfontein, have been transferred to the R.A.M.C. since the closing of the hospital, others have gone home or are doing duty at the Boer Concentration Camps. — An enjoyable evening was spent by the staff of the Grove Fever Hospital, Tooting, on Tuesday, the 11th inst., when the newly-formed minstrel troupe gave their first performance under the direction of Mr. T. W. Smith.

### Lectures to Nurses on Anatomy.

By W. JOHNSON SMITH, F.R.C.S., Principal Medical Officer, Seamen's Hospital, Greenwich.

### XI.—THE PELVIC GIRDLE (*Continued*)—THE BONES OF THE LIMBS.

THE pelvis, as a whole, may now be studied with regard to certain points of practical importance in obstetrical work.

*Obliquity of the Pelvis.*—The pelvis when occupying its natural position in the skeleton is not directed as it would be if detached and laid on a level surface. In its relative position to the spine it is not horizontal but oblique, its inclination downwards and forwards (fig. 24, B|C) forming an angle of from 140 to 145 degrees with a vertical line from the atlas to the upper part of the sacrum (A B), and an angle

FIG. 24.—Diagram showing obliquity of pelvis.

of from 50 to 60 degrees with a horizontal line (B D) drawn straight forwards from the promontory of the latter bone. In this natural obliquity of the pelvis the top of the symphysis pubis should be about four inches below the level of the sacral promontory.

On examining the pelvis from above, or in front, it will be seen that the large bony girdle at the level of the upper part of the sacrum behind, and of the top of the symphysis pubis in front, encloses a large heart-shaped, or rather kidney-shaped, space. This is the *inlet* of the pelvis, and its bony margin is called the *brim*. The part above the brim—that between the expanded wings of the iliac bones—is called the *false pelvis*, that below the brim the *true pelvis*.

The *outlet* of the pelvis is not so well defined in the skeleton, as its boundaries are formed chiefly by ligamentous structures. If the pelvis be turned "upside down," we may trace its outlines by carrying a string on either side from the lower part of the symphysis pubis to the tuberosity of the ischium, and then backwards and inwards to the tip of the coccyx.

*The Diameters of the Pelvis.*—A line drawn from the middle of the promontory of the sacrum to the symphysis pubis will measure the *antero-posterior or conjugate* diameter of the inlet, and a line measuring its greatest width will give the *transverse diameter*.

The antero-posterior diameter of the outlet is represented by a line drawn from the tip of the coccyx to the lower end of the pubic symphysis, and the transverse diameter by a line drawn between the ischial tuberosities.

Measurements of the diameters of the inlet will show that in a normal pelvis the transverse exceeds the conjugate; that is to say, the width of this opening exceeds its extent from front to back. With regard to the diameters of the outlet there is much uncertainty, and the teaching of some anatomists—that a reverse relation exists here and that the conjugate exceeds the transverse diameter—has not been confirmed by the results of recent and more extended investigation.

| *Diameters.* | *At the outlet.* | *At the inlet.* |
|---|---|---|
| Conjugate ... | about $4\frac{1}{4}$ inches | about $3\frac{3}{4}$ inches |
| Transverse ... | „ $5\frac{1}{4}$ „ | „ $4\frac{1}{4}$ „ |

It should be borne in mind that whilst the margins of the inlet are rigid and unyielding, the antero-posterior diameter of the outlet may on considerable pressure, such as occurs during parturition, be increased to some extent in consequence of the mobility of the coccyx, and so adapt itself to the direction of the long axis of the child's head.

*Sexual Differences.*—In the male, whilst the width of the shoulder girdle is greater, that of the pelvic girdle is less than that of the female. As Professor Thomson puts it, "women have broad hips and narrow shoulders, whilst men have broad shoulders and narrow hips." In the female the true pelvis—the part below the inlet—is more capacious than that of the male, the sacrum being less curved and the tuberosities of the ischium being turned outwards. An almost constant distinction of the female pelvis is the triangular contour of the large obturator foramen as compared with the oval form of this foramen in the male.

*The Limbs.*—The upper and lower limbs may each be divided into three segments—the upper one into arm, fore-arm, and hand; the lower into thigh, leg, and foot. In the thigh, as in the arm, there is a single bone; in both fore-arm and leg there are two long bones; in the hand there are as many as 27 bones, and in the foot just one bone less.

The bones of the corresponding limb-segments differ in size and shape. In the human skeleton there is this general distinction: the bones of the lower limb are larger, stronger, and more massive than those of the upper limb, as we might expect if we consider that the former support the weight of the body in rest and in locomotion, whilst the latter are used mainly for prehension.

The single bone of the arm, which is called the *humerus* (fig. 1, 4), has a long and straight shaft surmounted by a smooth rounded surface which is loosely attached by ligaments to

FIG. 25.—Upper extremity of left humerus. Front view.

FIG. 26.—Upper extremity o ulna. Side view.

the shallow "glenoid" pit on the shoulder-blade. Below it is expanded into a broad irregular extremity which forms the upper part of the elbow joint. Although the upper rounded extremity—the so-called head of the bone—forms less than half a sphere, and is set directly on the upper end of the shaft (fig. 25), two necks are recognised in the humerus—one the *anatomical neck* (A), corresponding to the margin of the cartilage which in the fresh bone covers the head; the other the *surgical neck* (B), situated below two processes of bone, the outer larger than the inner one, which may be observed on each side of a deep groove in front of the upper part of the bone. These prominent outgrowths of bone are called the *tuberosities* (C D), and the groove between them, which lodges a tendon, the *bicipital groove* (E). We must remember these two necks and their relative positions, as in ward work mention is occasionally made of fracture of the *surgical neck*, as distinguished from fracture of the *anatomical* or true neck of the humerus.

Of the two long bones of the fore-arm (fig. 1) that on the outer or thumb side is called the *radius* (6); that on the inner side, corresponding with the little finger, the *ulna* (5). The radius is a curved bone, thin at its upper extremity, which takes a very minor part in the composition of the elbow-joint, and thick and broad below, where it forms nearly the whole of the upper segment of the wrist-joint. The head, a round and concave disc at the upper extremity of the radius, surmounts a long and distinct neck, the junction of which with the shaft of the bone is marked by an osseous knob—the *bicipital tuberosity*—into which is inserted the tendon of the biceps muscle, which in muscular

FIG. 27.—Skeleton of wrist and right hand.

subjects is so prominent in front of the arm, and which, as it contracts, assists in bending forwards or flexing the fore-arm.

The *ulna* is quite straight and, moreover, differs from the radius in being thin and small at the wrist, and expanded above into a thick and broad extremity, which at the elbow forms nearly the whole of the articulating portion of the skeleton of the forearm. On this expanded upper extremity of the ulna are set two well-marked processes, a large one behind—the *olecranon* (fig. 26, A)—which clasps the posterior and lower parts of the humerus, and a smaller one in front, called the *coronoid* process.

At the lower extremity of each of the bones of the fore-arm there is a small pointed process of bone, which can be felt under the skin. These are the *styloid processes* of the radius and ulna.

The skeleton of the hand is made up by eight small and irregular bones in the wrist—*carpal bones*; five long bones imbedded in the soft parts of the palm—*metacarpal bones*; and fourteen bones, like in form but varying in size, in the thumb and fingers—*phalanges*.

The *carpal* bones are arranged in two rows, the first row in contact with the lower end of the radius, the second row articulating with the metacarpal bones. They are closely and firmly bound together by very stiong ligaments, and, together with these ligaments and opposed layers of cartilage, form a compact and composite buffer between hand and forearm, which is convex from side to side behind, and concave in the same direction in front. This set of bones, called *the carpus*, can be freely moved forwards and backwards on the lower extremities of the radius and ulna; but below, whilst loosely attached to the thumb, so as to allow a wide range of movement to this member, is closely bound to the long bones of the fingers.

The eight bones of the carpus (fig. 27) vary in size, and are, with the exception of the smallest bone, very irregular in shape. To be able to distinguish any individual and separate bone, it is necessary to study a complete set of loose carpal bones. These, if arranged in their proper relative positions, will form two rows of four bones each. In following the first and upper row from the side of the thumb to the side of the little finger we observe (1) the *scaphoid* or boat-shaped bone; (2) the *semilunar* bone, so called from its supposed resemblance to a half-moon; (3) a wedge-like or pyramidal bone, which has recently changed its well-known and appropriate name of "cuneiform" for the name of "pyramidalis" and (4) a small and rounded bone which, from its resemblance to a pea, is called "the pisiform." In the second and lower row, taking the same direction from thumb to little finger, we see (1) the *trapezium* and (2) the *trapezoid* bones, so called from supposed resemblances to these geometrical forms; then (3) the *os magnum*, or large bone; and, finally, the *unciform* or hooked bone, which sends off from its front surface a distinct hook-like process.

The next series consists of one metacarpal bone corresponding to the thumb and one to each of the fingers. Each of these metacarpal bones has a well-marked head at its lower extremity, and a square upper extremity called the *base*. Of the five bones, the first is the shortest and the second the longest.

Of the next and terminal set of bones (phalanges), three are distributed to each finger and two only to the thumb. The bones of each finger diminish in size from above downwards, the third being much smaller than the first.

If we seek in text-books on anatomy for some explanation of this supposed disappearance of a phalanx in the thumb, we shall find that it is really not a phalanx that is missing, but the metacarpal bone, the so-called first metacarpal bone differing from the other four metacarpal bones, and resembling the phalanges in its mode of development.

### Death in Our Ranks.

"NURSE OPHTHALMIC."

WE regret to announce the death, after 20 years of devoted work, of Miss Annie Brewster, which took place at the London Hospital, on Tuesday, February 11th. Miss Brewster, better known to her friends as "Nurse Ophthalmic," was trained at the London Hospital, and spent all her nursing years there, being the greater part of the time in charge of the ophthalmic ward. She was actively engaged in her work up to within a fortnight of her death. The funeral took place from the hospital, a very unusual occurrence, owing to the fact that Miss Brewster's relatives live abroad. She was buried at Ilford on Friday last. Just a fortnight before Miss Brewster's death, the hospital lost another devoted nurse in Miss Loweth, who had been at the hospital nearly 17 years. Both will be greatly missed, as they were two of the oldest workers at the hospital, and were valued and admired by all, while those of the nurses who knew them more intimately have lost in them personal friends.

### The Nurses of St. Pancras Infirmary.

A CHAT WITH THE MATRON: BY OUR COMMISSIONER.

The situation of St. Pancras Infirmary is entirely in its favour, for, though the north wind may be somewhat too keen in winter on the summit of Dartmouth Park Hill, the bracing air must be beneficial to the inmates. So far as the nursing staff are concerned, they appreciate—as Miss Moir, the matron, told me on the occasion of my visit—the proximity of the park, and regard it as a compensation for the distance of the infirmary from the heart of the Metropolis. The exterior of the building is not attractive, but the wards are roomy, lofty, and light. They are, apparently, exceedingly well cared for, and there is an air of cheerfulness even about some of the patients whose sufferings are obvious, which creates a pleasant impression. I was agreeably surprised at the size and the comfort of the recreation room in the Nurses' Home, and, though economy is evidently studied in the establishment, there are no indications that essential wants are overlooked. It would save the feet of the nurses and facilitate work if lifts were introduced, and there must be times when the need of a covered way in getting from one part of the building to another is experienced. The Infirmary was the first of its kind erected after the passing of the Metropolitan Poor Act 1867, was transferred to the managers of the Central London Sick Asylum District in September, 1870, and was bought back again by the St. Pancras Guardians, March, 1883. Having got to this point in historical matters, I asked Miss Moir to tell me about her own associations with the infirmary.

"I am the first matron," she said, "under the St. Pancras Board, after the transfer of the Infirmary. The Sick Asylum, as it used to be, was, in succession, under the charge of two matrons who came from the Nightingale Home. The diagrams for training probationers which had been sent from the Home were in use when I took up the duties here."

"You did not come from the Nightingale Home?"

"No; I was trained at Edinburgh Royal Infirmary, and had the privilege of working in both the old and the new buildings. From Edinburgh I went to Westminster Hospital as a member of the private nursing staff under Miss Pyne, who was then superintendent. Afterwards I was night sister at the London Hospital."

"Have you done any district nursing?"

"I always wish I had, and also that I had taken the L.O.S. certificate, though I think that private work is the most important branch which a nurse can take up after she leaves the hospital. It teaches patience, and is a help in the way of enabling one to form an accurate judgment."

### The Commencement of Training.

"How many probationers did you start with?"

"Six only. At that time the infirmary was managed on the usual Poor Law system. There was a charge nurse in every ward of 32 patients, and a ward assistant. The first progressive step was to increase the probationers to twelve. But the great change came eight years ago, when we did away with the ward assistants and employed scrubbers from outside and made room for 50 probationers and nurses.

"Is that the present number?"

"Yes, the entire nursing staff now consists of 17 first year probationers, 33 staff nurses, 7 sisters, 3 assistant sisters, 2 night sisters, and an assistant matron. No certificate is given for less than three years' training. In their second and third years the probationers do staff nurses' duty day and night work alternately—four months day, and four months night."

### Pay and Instruction.

"Have you any difficulty in filling vacancies?"

"Well, we just manage to fill them. The fact that we do not give any salary for the first year hinders some from applying. We pay £15 the second year and £20 the third."

"And uniform?"

"Both indoor and outdoor uniform are given. We expect the nurses to wear uniform out of doors, and, as a rule, they like to wear it, especially when their time is limited."

"What about the instruction?"

"Lectures are given by the medical and by the assistant medical superintendents, and classes by the matron, the assistant matron, and the night sister. There is an outside examiner — Mr. Mower White, of Harley Street — who examines twice a year."

"Do the nurses pass as a rule?"

"Yes; and those who do not are allowed to go in for another examination. They are very nervous—which, after all, is natural enough, if they feel that their whole future may depend upon their success or failure at the examination."

### Hours and Diet.

"How long are the hours of duty?"

"From 7 in the morning to 8.30 at night. But two hours off duty are allowed; and of the remainder, three-quarters of an hour for lunch and dressing, half an hour for dinner, and half an hour for tea. A day and a half off are given in the month, and three hours on Sunday. The hours of the sisters are the same as those of the probationers; but while the latter have 16 days' holiday the first year, and 21 days' the second and third years, the former have a month. If we had more sisters, I should like to shorten their hours. The night sisters have two nights off every month, and the night nurses one."

"What are the hours on duty at night?"

"From 8.30 P.M. until 8 A.M. The night nurses dine at 8.30 A.M., and are out of doors from 9 until 12. I was much interested in the letters on the diet of night nurses in The Hospital. Here it is just the same as that of the day nurses, and I think that the food is excellent. It is varied as much as possible."

"You might give me an idea of the nature of the diet."

"I give the nurses always a pudding for dinner and a pudding for supper, soup twice a week, and fish twice a week. We try to vary the puddings as much as possible, and six days out of seven fruit is provided—either an orange, an apple, a banana, or other fruit in season. Of course, we are on rations, but the system can be worked very well if sufficient care is taken.

### The Nurses' Home.

"When was the Nurses' Home added?"

"In 1894, at the time we increased the number of probationers. There is sleeping accommodation in the home for 34. Each nurse has a separate room. The night nurses all sleep in the home."

"And the remainder of the staff in the administrative building of the infirmary?"

"Yes. We are so cramped for space that in some cases three sleep in a room. All the staff except the night nurses have their meals in the home, and the latter have breakfast and dinner there. The meals during the night are taken in the little ward kitchen. The assistant matron is in charge of the home."

"I see that a piano is provided."

"Yes, one was kindly given by a lady guardian. There is a tennis lawn for outside recreation."

"How many nurses are allotted to each ward?"

"There are six nurses and a sister on duty during the day in a three-ward block; and four nurses and a sister in a two-ward block. We have five three-ward and two two-ward blocks. There is one night nurse to each ward of 33 patients, and two night sisters for the whole infirmary."

"I notice that there are children in most of the wards."

"In every one. We have a children's ward for 32 cases, but many children being unfit for various reasons to mix with others, these have to be placed in the adult wards."

VACCINATION IMPERATIVE.

"The question of the re-vaccination of nurses is attracting a good deal of attention just now, owing to the fact that at an East-end infirmary so many have contracted small-pox. Have all yours been re-vaccinated?"

"Not only the nurses, but every employee in the building, including the outside scrubbers. Some of the patients have also been done, but many refuse. Every one of our staff was re-vaccinated as quickly as possible after the first case of small-pox occurred in St. Pancras."

AFTER TRAINING.

"When your nurses have finished their training do they generally continue to serve under the Poor Law?"

"No, most of them go in for private nursing. Some become district nurses either in Scotland, London, or the provinces. One or two have gone into general hospitals. But before they take up either of these branches of work they almost invariably go either for a short term of fever training to one of the hospitals under the Metroplitan Asylums Board or to one of the maternity hospitals. Some of our old probationers are now on the Nurses' Co-operation; two went out to India to do plague nursing, one of whom, I regret to say, died; another accepted a post in Egypt, and yet another went out to West Africa."

PROVIDING FOR THE RAINY DAY.

"Do any of the present staff belong to the Royal National Pension Fund?"

"One or two. Of course many contribute to the Poor Law pension fund. They pay 2 per cent. on their salary and emoluments, and after ten years, if they are unfit for work, they get a sixtieth of their salary and emoluments for every year. But they get nothing unless, or until, they have served for ten years."

"The principle," concluded the matron, "is altogether different from the National Pension Fund, whose subscribers can withdraw the amounts they have contributed. I was one of the first to join the National Pension Fund, and I have always considered it an excellent institution. It would merit support, if only because it teaches nurses how to put by for a rainy day."

### Fever Nursing in its Relations to Fever Hospital Administration.

By J. T. C. NASH, M.D.Edin., D.P.H.Camb., late Demonstrator in Bacteriology, King's College, London; Medical Officer of Health, and Medical Superintendent Borough Sanatorium, Southend-on-Sea; Resident Physician, Brighton Fever Hospital; Lecturer on First Aid and Home Nursing, London School Board.

*Concluded from page 259.*

(3) We turn next to diseases like measles, scarlet fever, small-pox, or chicken-pox. In these maladies, the bacteriology of which is still uncertain, we have every reason to believe the germ exists in a virulent condition, not only in all the mucous membranes and their excretions, but in the external skin and its secretions and excretions These diseases are characterised by a distinctive rash, followed by desquamation, and the chief danger in the desquamation is that the germs borne on the skin scales are like voyagers on miniature rafts in the air-ocean carried about by every slight draught or breath of air. In all of them the germs also exist in the air passages, and therefore cough and sneezing and even talking are dangerous if any susceptible person comes within range. In these infectious diseases, then, extra special precautions are necessary. Not only must the nurse or attendant exercise the usual precaution as to her clothing and hands, but the cases must be effectively and rigorously isolated. The small-pox contagion appears to be carried further and more mysteriously than that of any other disease, and therefore there can be no doubt as to the necessity of special small-pox hospitals. The organic cause of small-pox has not yet been discovered, but it is highly probable that it is so extremely minute that it cannot be recognised even with the powerful microscopes that are now made. I believe it is so minute and light that the slightest current of air, such as is induced by the mere presence of a warm animal body in a room and which is quite imperceptible even to a delicate instrument is sufficient to waft it away. Currents of air which would not disturb even such minute particles as ordinary bacteria, might conceivably be sufficient to carry so extremely minute a germ as that I have hinted at, a considerable distance. If this be so, the inadvertent introduction of a case of small-pox into any ward must necessarily be attended with the gravest risk in spite of all ordinary or special precautions. In a less degree, perhaps, the above remarks might be applied to *chicken pox*, but, fortunately in this disease, the organism is not so virulent. Coming now to deal with *measles*, we are considering an extremely infectious disease, the most dangerously infective period being probably in the early stages before the rash appears. Any case with nasal discharge or watery conjunctivæ or cough should be considered by the nurse to be a case requiring every precaution on her part as to disinfection of hands and change of outer garments before attending to another case.

The bacteriology of measles is still uncertain, but apparently the pathogenic organism is principally to be found in the mouth and nose, and consequently discharges from these require very special attention, and sneezing and cough are particularly to be feared. In such cases mischief might very well result before the doctor sees the patient, and therefore I particularly commend the system adopted in certain French hospitals at present, which consists practically in isolating the patient even in a general ward by means of glass screens which are arranged all round the bed at a sufficient height to arrest particles of mucus or drops of saliva which may be ejected with a sneeze or cough. A special cloak can be kept in this little cubicle for the nurse to put on when she goes in to attend the patient, and is to be removed again on leaving, and left behind in the cubicle, the hands being then soaked in a disinfectant. The food utensils must also be sterilised in the cubicle before removal. The floor and glass panels must be daily washed. Experience in France has shown that in this way, exercising every precaution, measles may be treated even in a general ward without spreading. It is obvious, however, that this can only be achieved if the nurse thoroughly understands what precautions she must take, and thoroughly and conscientiously carries them out. Good nursing essentially consists in intelligent comprehension and conscientious act.

The same precautions would probably prove equally effective in cases of *whooping cough*, another disease of uncertain bacteriology, but in which there is no doubt the infection is carried by the breath in the acts of coughing, and sneezing, and vomiting. Doubtless similar precautions would avail in the case of an outbreak of scarlet fever in a ward reserved for other cases, and should certainly be resorted to until the physician has seen the suspected child. Of course a separate ward and separate nurse, for each suspicious case, would obviously meet the difficulty, but it is equally obvious that this is quite impracticable, and, humanly speaking, impossible. As an alternative, the method I have mentioned has everything to recommend it, but it would depend almost entirely on the intelligent and conscientious co-operation of the nurse whether it would prove workable or not.

The object of this article is not to advocate the treating of infectious diseases in general hospitals, nor the treatment of two different infectious diseases in a common ward in a fever hospital. There can be absolutely no question as to the advisability of having special fever hospitals, with separate blocks, for each disease. But all who have had to do with fever hospital administration know that cases are not infrequently admitted with a second infective disease incubating or actually present. It is for this reason that I recommend that every new case which might unavoidably be admitted during the absence of the doctor should be dealt with as a suspicious case until seen by him, and that the nurse, who is trained to be on the *qui vive* for unusual symptoms, should, when any such develop, at once take extraordinary precautions, even prior to the doctor's visit, for even though these precautions may have been unnecessary, they can never do harm. On the other hand I am inclined to believe they will almost invariably prevent an outbreak of a second disease in the ward if they are carried out early in a genuine case of double infection.

### A German Hospital Kitchen.

BY AN IRISH NURSE.

FINDING myself in the old university town of Heidelberg during carnival time, when the streets are filled with merry masqueraders, I accepted an invitation to see the great hospital kitchen at work on Shrove Tuesday. Owing to the fame of the medical and surgical staff, the hospitals, of which there are eight or nine built along the River Neckar, attract patients from far and near, and are therefore on a much larger scale than the town itself requires. Patients are divided into classes, the first and second paying 7s. to 10s. daily, having private, well-furnished rooms and first-rate food, the third—and naturally by far the most numerous—class is paid for by the Kasse, or Insurance Society, to which the patient belongs, or by his native town or village if he comes from a distance.

Owing to this division of patients cooking is more elaborate than in most English hospitals, for the patients in the first class on full diet get for dinner soup, two courses of meat, and vegetables and sweets, served as they would be in an ordinary hotel.

The kitchen I visited cooks only for the medical, surgical, and eye wards of the University Hospital, containing about 450 patients, and with a staff of 180. Of these 14 are in the kitchen, headed by a chef and presided over by a lady housekeeper. The feasting of carnival time outside was reflected in colossal stacks of pancakes, fortunately of a lighter make than ours, as they were being turned out on the supposition that each patient would eat four! and no doubt the 22 resident doctors and the nurses had no intention of falling behind the patients. Fried a delicate golden brown, crisp and delicious, I could understand that four was not an impossible average. Sugar is sprinkled over them, and they are served with stewed fruit. In the making of these pancakes 160 lbs. of flour had been used, besides large quantities of eggs, sugar, butter, and milk.

### "The Hospital" Convalescent Fund.

THE hon. secretary acknowledges with thanks the receipt of 2s. from Miss E. M. Geach.

### Appointments.

[No charge is made for announcements under this head, and we are always glad to receive, and publish, appointments. But it is essential that in all cases the school of training should be given.]

BRIDGEWATER INFIRMARY.—Miss Mary F. May (not Way, as inserted last week), has been appointed matron.

CUCKFIELD ISOLATION HOSPITAL.—Miss M. A. Plough has been appointed matron. She was trained at the Royal Surrey Hospital, Guildford, and has since been charge nurse at the South-Eastern Fever Hospital, New Cross; matron at Leighton Buzzard Sanitary Hospital, and matron at the Infectious Diseases Hospital, Darwen.

DAGENHAM ISOLATION HOSPITAL.—Miss Florence F. Fyers has been appointed night superintendent, and Miss Louise L. M. Payne has been appointed assistant nurse. Miss Fyers was trained for three years at the Birmingham Infirmary. She holds the L.O.S. certificate. Miss Payne was trained at the Borough Fever Hospital, Ipswich.

GENERAL INFIRMARY, CHESTER —Miss Edith Stansfeld has been appointed home sister and assistant matron. She was trained for three years at St. Mary Abbots Infirmary, Kensington, and has since been ward sister for two years in the same institution. She has also been charge nurse for two years at the Grove Fever Hospital, Tooting, where she took holiday duties for the assistant matron and night superintendent; and district nurse for a year at the Ormond Home, Chelsea. Miss Stansfeld holds the L.O.S. certificate.

GORE FARM HOSPITAL, DARTFORD.—Miss Alice Meadows has been appointed assistant night superintendent. She was trained at East Dulwich Infirmary, and has since been engaged in private nursing at Eastbourne; head nurse at the Royal Hospital for Incurables, Putney; and charge nurse at the Grove Hospital, Tooting.

GRAVESEND AND MILTON WORKHOUSE INFIRMARY.—Miss Florence Gorman has been appointed nurse. She was trained at Uxbridge Joint Hospital.

MERCER'S HOSPITAL, DUBLIN.—Miss Juliet Collier has been appointed sister of the male surgical ward and theatre. She was trained for three years at the Norfolk and Norwich Hospital, where she subsequently became assistant nurse. She was also in charge of the surgery and out-patients' department. On leaving the Norfolk Hospital she joined the Birmingham District Nursing Society.

MOUNT VERNON HOSPITAL FOR CONSUMPTIVES, HAMPSTEAD AND NORTHWOOD.—Miss Marian Measures has been appointed matron. She was trained at Guy's Hospital from October, 1886, to November, 1889. She has since been ward sister at Kent and Canterbury Hospital, April, 1890, to April, 1891; night sister Manchester Children's Hospital, Pendlebury, May, 1891, to September, 1891; ward day sister in the same institution, September, 1891, to July, 1896; assistant matron and home sister at the Seamen's Hospital, Greenwich, July, 1896, to August, 1899; and matron at Gravesend Hospital from August, 1899, to the present date.

OSWESTRY ACCIDENT HOSPITAL.—Miss Emily F. C. Eastcott has been appointed staff nurse. She was trained for three years at the Seamen's Hospital, Greenwich, and the Women's Hospital, Soho Square, London, afterwards receiving district training at St. Patrick's Home, Dublin. She has since been nurse at the Meath Hospital and County Dublin Infirmary, and for some years has been attached to Queen Victoria's Jubilee Institute.

RYDE COUNTY HOSPITAL.—Miss Bateman has been appointed night sister. She was trained at the General Hospital, Nottingham, and has since been staff nurse for two years at the Children's Hospital, Pendlebury, Manchester.

ST. PETER'S HOSPITAL, COVENT GARDEN, LONDON.—Miss M. C. Morrison has been appointed sister. She was trained at Arbroath Infirmary. She has since been nurse at the Royal Hospital for Sick Children, Edinburgh, and sister at the Grantham Hospital.

SOUTHAMPTON WORKHOUSE INFIRMARY.—Miss E. M. Parsloe has been appointed assistant matron. She was trained at Kensington Infirmary for three years, has since been sister at St. Pancras Infirmary, and sister of surgical wards at the County Hospital, Bedford. She has also done private nursing.

STOCKTON AND THORNBY HOSPITAL.—Miss F. M. Bates has been appointed matron. She was trained at the Royal Infirmary, Newcastle-on-Tyne, and has since been head nurse at Darlington Hospital, for three and a half years, and matron of Louth Hospital for two and a half years.

UP-COUNTRY NURSING ASSOCIATION FOR INDIA.—Miss Morten has been appointed nurse. She was trained at Hampstead Hospital, Parliament Hill, London. She has since been staff nurse at St. Mary's Hospital, Paddington, and has been engaged in private nursing.

### Everybody's Opinion.

### AN APPEAL FROM MEALSGATE, CARLISLE.

"RUTH DRAKEFORD," Ireby, Mealsgate, Carlisle, writes: In my appeal for certain articles for the use of my patients in this district my surname was not printed. Hence Lady Gillford's letter last week. I have nothing whatever to do with Lady Gillford or the Cumberland Nursing Association, but work under a committee which is called the "Ireby, Elldale, and Bolton Gate District Nursing Association." I have a great many poor patients and shall be happy to receive anything useful for their comfort which will be gratefully acknowledged.

### "B.-P. AND THE NURSES' CLUB."

"EMILY MACLEOD," nurse at the Royal Infirmary, Dundee, writes:—It was with feelings of great pleasure I read the note in your issue of February 1st entitled "B.-P. and the Nurses' Club." I now take the opportunity of informing you that about eighteen months ago the nurses on night duty in the City Hospital, Edinburgh, formed a rambling club, naming it the Baden-Powell Rambling Club. Being appointed secretary, I had the honour of writing to General Baden-Powell. The letter was sent enclosed in one from our matron, Miss Sandford, but no answer was received. Although most of the original members are now in different institutions, it gives me much satisfaction to acquaint them with the fact that our letter was received. Should our club be the one referred to, I would consider it a great honour to receive any communication regarding it.

### OVERWORKED MALE NURSES.

"H. W." writes: For 17 years I have been a male nurse and have worked under some of the best doctors in London and other large towns. I have been trained in hospital and asylum. Of late I have been disgusted at the number of men there are who call themselves "attendants" and are in reality only servants, although they are kept because the master of the house is an invalid. They valet, draw a bath-chair, and do the duties of footman, butler, and, in fact, all kinds of domestic work for the small sum of £20 to £30 a year as wages. When the employer is taken ill they have to send for another man who is a trained nurse, and when he asks £2 2s. a week the family think it a shame to pay so much. A year ago I nursed a gentleman through a serious illness, and when he got better and was able to go out in a bath-chair (although not able to attend to himself) I was sent away and a man came in my place to do my work for £20 a year. He had also to do garden work and house work as well, and he is doing it still. I have refused many places because I have not been willing to undertake to make myself useful, which would mean doing all the work of a common odd man. But why do not these people who employ a man under the disguise of a male attendant pay a tax?

### WORKHOUSE NURSING.

"LOUISA TWINING" writes: I am glad to find that this subject continues to be discussed in your columns, and the recent appointment of a committee of the Local Government Board to consider it, is most gratifying to those who for long years have been urging reforms. I venture to send for your insertion a few remarks by a former experienced chairman of an important country Union as a valuable confirmation of what I have repeatedly suggested as necessary and desirable. He says, with regard to the proposed classification of workhouse buildings, "I think the scheme is a capital one; unless something of the kind should be adopted, I do not see how it is possible that our country workhouse infirmaries should be what they ought to be. The one I belonged to might be converted into a district hospital. In starting it as such, the initial expense would be, of course, the great difficulty, but no one can say what may be done if a persistent and vigorous effort is made. Then, there must always be friction in workhouses till the masters and matrons are taken from a higher social class. They are so in prisons, and they ought to be so in workhouses; the latter are quite as hard to manage properly. But here again a sufficient salary would be the difficulty." It is at least 40 years ago that I urged these necessary points of reform, and till they are carried out, the only way to ensure peace and good management will be to separate as far as possible the care of the sick, under the control of the head nurse and medical officer, but the plan for a central workhouse of a union for the sick alone, under trained nursing, is, I believe, the only real solution of all our difficulties.

### Presentations.

ST. LEONARD'S INFIRMARY, SHOREDITCH.—A very handsome silver manicure set was presented to Miss Appleyard by the doctors, sisters and nurses of St. Leonard's Infirmary Shoreditch, last week, on the occasion of her resignation of the post of assistant matron, which she has filled so ably for the last three years. Miss Appleyard's resignation has caused many regrets, for she has done much to raise the standard of the nursing in the infirmary.

THE HOSPITAL CONVALESCENT HOME, PARKWOOD, SWANLEY.—On leaving this establishment Sister Preston has been the recipient of many useful and valuable presents. Mr. Peter Reid (the founder), the trustees, and the chaplain of the home each presented her with a cheque; the senior officers and Mr. and Mrs. Staples gave a carriage clock; the nurses, a silk umbrella with a silver-mounted handle and a handsomely bound book; the servants, a brass kettle on stand. Sister Preston has been six years at the home, and is leaving to take up the duties of night sister at St. Mark's Hospital, City Road, London.

### Wants and Workers.

WOULD anyone kindly send THE HOSPITAL on the Monday after publication, on payment of three months' postage in advance? Address, Mrs. Moore, Messingham, Brigg, Lincolnshire.

A PARISH nurse would be grateful for THE HOSPITAL sent to her the Monday after publication, postage prepaid for six, months. Nurse Annie, 24 Park Road, Mexboro', Rotherham, Yorks.

### TRAVEL NOTES AND QUERIES.

CORK AND BLARNEY (Sister Rachel).—The cheapest way is by Bristol, first return 22s. 6d., second return 11s. 6d.; second-class is quite comfortable. The steamers go twice a week from the Cumberland Basin; second return from London to Bristol, 21s. 9d. The next cheapest way is *viâ* New Milford, second return £2 15s. Blarney is a small place nine miles from Cork; there is one small hotel near the station. I have never stayed there, though I think it might be comfortable; but Blarney is not so interesting as Glengariff, Killarney, etc. . . . You would find information useful to you in an article I wrote in *Home Notes*, for the week ending August 8th, 1901, called "A Trip to Ireland from the West of England." Write to the editor for it at Henrietta Street, Covent Garden. Length of journey from Bristol about 12 hours, I believe.

### Off-Duty Letter.

### AMERICAN DRAMA AT THE ADELPHI.

You will have seen that the King and Queen, with two of their daughters and their son-in-law, visited the Adelphi on Friday and sent for Miss Olive May at the end of the piece to compliment her. I too went the other evening to see "Arizona," and as I returned home I reflected that English managers might with advantage take a couple of hints from Mr. Kirke La Shelle's company. First, as to the programme. Most of us have suffered at some time from an inability to sort out early in the play the different characters, so that we were somewhat bewildered as to the various *dramatis personæ* till the piece was half finished. No trouble of this kind can occur under present auspices at the Adelphi, because the names of the characters are given in the order in which they make their appearance on the stage, with of course the necessary explanation as to their relationship to each other. Again, when at the end of any of the acts the curtain is raised in answer to the applause of the audience, there is something better to be seen than a long line of successful actors and actresses bowing and smiling with a pleased air of smug satisfaction. When I was at the theatre the curtain was run up six times in succession after the third act, and each time the action had progressed a step farther. On the first occasion the wounded man had been raised from the floor, and the doctor was endeavouring to get his coat open; on the second "first aid" had been rendered with the help of the Red Cross nurse, and the ambulance was being brought in; on the third the invalid had been lifted on to the ambulance and the bearers were taking him away. Thus the story continued in tableaux as it were—the other actors taking up their parts in the same natural manner—until gallery and stalls alike were pacified.

As to the play, it is an excellent melodrama, with plenty of plot, plenty of excitement, and plenty of fun, with a hero who is not a prig, a heroine who is a very winsome lassie, and a thorough-paced villain quite bad enough to let the "gods" feel justified in hissing him whenever he comes on the stage. The initial act is a little difficult to follow. The American accent, which most of the actors possess, and which from the surroundings of the piece they would be justified in acquiring, sounds strange to English ears, and the military commands are necessarily given rapidly. This, together with the quick pace at which the action proceeds, makes the whole at first somewhat difficult to understand. But the inconvenience is only temporary, and there is ample time left for enjoyment. The story is somewhat complicated. Henry Canby, the rich owner of a ranch, has two daughters. The elder, Estrella, is married, after love passages with a Captain Hodgman, to Colonel Bonham, of the 11th U.S. Cavalry, a man much older than herself. The younger, Bonita, is a merry, happy little soul, courted, amongst others, by Lieutenant Denton.

There is a charming bit of lovemaking in the first act, where a couple of side-combs from the lady's hair play an important part; the downrightness of the lover and the coy archness of his beloved are specially pleasing. We are also introduced to a maid-servant, Lena Kellar, "with a past," and to a most picturesque Vaquero, Tony, who is fast losing his heart to her. In the second act, where the scene is removed from Canby's ranch to the military quarters at Fort Grant, the Colonel is called away after a dance. He bids farewell and departs, leaving his wife free to elope with Captain Hodgman, as previously arranged. Denton, who is devoted to the Colonel, gets wind of the scheme, frustrates it, packs off the scheming Captain, only to be discovered by the Colonel—who has unexpectedly returned—at 3 A.M. in his wife's rooms. In addition, Mrs. Bonham's jewels are found upon the young Lieutenant, and, it being known that he has lost money at poker, he is accused of theft, and would have been dismissed the army if he had not resigned.

All this time the wife, who knows the true circumstances of the case, stands by and says nothing, but weeps copiously. In the next scene we are back again at the ranch. Denton is headman to Canby, and on the high road to become his son-in-law. He would not have asked Bonita to be his wife, because of the disaster which had befallen him; but she makes such a sweet, womanly appeal to him that he feels bound to speak out. The ranch owner is delighted, promises Denton shall be part owner, and then Hodgman appears on the scene. He tries to poison the minds of Canby and his wife against Denton, who, infuriated, strikes him across the face, and at the same time pulls out his pistol. At that moment a shot is fired and Hodgman falls. Denton is arrested for the crime, which has in reality been committed by the Vaquero, who has learnt from Lena that it was Hodgman who was her betrayer. The last act is the trial of young Denton. All goes against him until the bullet is extracted from the dying man's chest, and it is found that it will not fit Denton's pistol. Lena is then accused, and to save her Tony confesses; but clearing a space to show just how he fired, he manages to rush through the open door, springs on to a noted swift horse, and escapes. His chance of getting off scot free is a good one, for the Colonel tells his men to chase him but forbids them to fire. The Lieutenant, now happy himself, pleads that the Colonel will not be too hard on his wife, who has made a full confession, and pressing a rose to his lips which she has worn at her neck the elder man goes off to the Cuban War, promising to try and forgive when he returns—a much more rational proceeding than the "Kiss and be friends" which I feared was coming to spoil the artistic finale.

The author of the drama, Mr. Augustus Thomas, is fortunate in having a capital company to interpret his characters. From the most important to the least, each does his and her utmost for the part entrusted to them, and the harmony of the whole is complete. The honours fall particularly to Miss Olive May and Mr. Vincent Serrano. Miss May has the gift of a very expressive face, which reflects, as she means it to do, every thought and fear; and this, united to a singularly sweet voice, does much to make her the charming little actress she is. Her roguery is delightful, but she is not less successful in the more pathetic parts, and Harry Denton, as portrayed by Mr. Vincent Serrano, is a hero any girl would find it easy to be nice to. The young man—I do not know if he is as young as he looks, but he has almost a boyish appearance—has a fresh and breezy manner, which leaves no doubt of the sincerity of his intentions. He holds himself well in hand, and seldom allows his feelings to run away with him, so that when occasionally he lets himself go it is the more "telling." Mr. Edgar Selwyn, as Tony, looks exceedingly picturesque, and acts with considerable strength. The impulsiveness of the southern character is well portrayed, and he sings a Spanish love song with much taste at the beginning of the third act. Mr. William Harcourt is dignified and handsome as the grey-haired Colonel, Mr. Theodore Roberts jolly and loud-voiced as the owner of the ranch, and Miss Eleanor Wilton gives due emphasis to the rather shrewish character of his wife. Miss Mary Hall does what she can for the unloving wife, whose chicken-hearted behaviour causes all the mischief, Mr. Joseph Kilgour makes love as hotly and hates as strongly as the orthodox villain should, and Mr. George O'Donnell gains a good deal of applause as the happy-go-lucky good-tempered surgeon of the regiment.

### A Book and its Story.

R. L. STEVENSON'S STUDIES.*

To those readers who appreciate and delight in Louis Stevenson the present reprint of "Men and Books," in a bijou two-shilling edition, will be very welcome. Print, binding, and size, all combine to make it a charming possession possible to the owner whose taste for literary gems is out of proportion to his power of gratifying it otherwise than at a reasonable cost. The "studies" included are varied enough. They are those of men of world-wide reputation, Victor Hugo, Thoreau, John Knox, Burns, Walt Whitman, François Villon, and others, chosen by the author as being "men whom for one reason or another I loved; or when I did not love the men my love was the greater to their books. I had read them and lived with them; for months they were continually in my thoughts; I seemed to rejoice in their joys and to sorrow with them in their griefs."

The characters of Knox and Burns are considered by the author as being most difficult of comprehension by an outsider. As their countryman no one, perhaps, was better able to write an illuminative criticism than Stevenson himself. He says, "to pass true judgment upon Knox and Burns implies a grasp upon the deepest strain of thought in Scotland—a country which differs far more from England than do many parts of America; for, in a sense, the first of these men re-created Scotland, and the second is its most essentially national production." As so many readers of The Hospital are women leading independent, self-supporting lives, the chapter on John Knox's private life and his relations to women will be read with interest and amusement. "To those who know John Knox by hearsay only, I believe the matter of this paper will be somewhat astonishing . . . he remains for posterity in certain traditional phrases—brow-beating Queen Mary, or breaking beautiful carved work in abbeys and cathedrals that had long smoked themselves out and were no more than sorry ruins while he was still quietly teaching children in a country gentleman's family. . . . He had considerable confidence in himself and in the underlying uprightness of his disciplined emotions, with much sincere aspiration after spiritual humility. And it is this confidence that makes his intercourse with women so interesting to a modern. It would be easy to make fun of the whole affair, to picture him strutting vaingloriously among those inferior creatures, or compare a religious friendship in the sixteenth century to what was called, I believe, a literary friendship in the eighteenth. . . ." Early in 1558 he had published, as a protest against the advance of the sex in high places, his notorious book "The First Blast of the Trumpet against the Monstrous Regiment of Women," and with full consciousness of a great duty before him he set himself to answer the scruples of timorous or worldly-minded people. How can a man (much less a woman) repent, unless the nature of his transgression be set plainly before him? "And therefore I say," he continues, "that of necessity it is that this monstriferous empire of women (which among all enormities that this day do abound on the face of the earth is most detestable) be openly and plainly declared to the world, to the end that some may repent and be saved." Among his correspondents we are told of a little group known as his "Sisters in Edinburgh," and "It must not be forgotten that Knox had been a presbyter in the old Church, and that the many women who gathered around him had probably been accustomed, while still in the communion of Rome, to rely much upon some spiritual director." This probably explains much, but his "Sisters in Edinburgh" had to "provoke" his attention pretty constantly, and the matters on which they sought counsel on some occasions were hardly such as to extract consolation from the Knox. Women's dress, for instance, was one. He exclaims: "The garments of women do declare their weakness and inability to execute the office of man." Although he deprecated the claims of the New Woman as being too masculine, yet, on the other hand, his "Sisters in Edinburgh" seem clearly to have bored him at times; he found them weak, frail, impatient, feeble, and foolish. Knox, in spite of the monstrous plague of women, was twice married. His wives probably were each taken from the two types and formed the text which inspired the discourse on the "Monstrous Regiment." After the accession of Elizabeth he found it politic to moderate his denunciations, and we read that "he perceived the *Blast* had been blown out of season, and the Regiment of Women was one of those imperfections of Society which must be borne with because yet they cannot be remedied."

Henry David Thoreau, the American philosopher and naturalist, born in 1817, has an interesting appreciation. Thoreau began life as a schoolmaster and drifted into that of an ascetic. Upon the author "this pure sunnily ascetic Thoreau" exercised a great charm. "Now, Thoreau's tastes were well defined. He loved to be free, to be master of his times and seasons, to indulge the mind rather than the body: he preferred long rambles to rich dinners, his own reflections to the considerations of society, and an easy, calm, unfettered active life among green trees to dull toiling at a desk. Prudence, which bids us all go to the ant for wisdom and hoard against the day of sickness, was not a favourite with Thoreau. He preferred that other, whose name is so much misappropriated, Faith." So Thoreau retired at twenty-eight to lead the better life. He went out into the woods, built himself a hut, and made friends with Nature as found in his immediate neighbourhood. "The hunted fox came to him for protection; wild squirrels have been seen to nestle in his waistcoat; he could thrust his arm into a pool and bring forth a bright panting fish." There were few things that he could not do. He could write books that are still read with delight, notably his charming "Walden." He was a scholar, a surveyor, and natural historian. The following expression, taken from his writings, suggests something of the inner man: "Why need we go abroad, even across the way, to ask a neighbour advice? There is a nearer neighbour within, who is incessantly telling us how we should behave. But we wait for the neighbour without to tell us some false, easier way."

Yoshida-Torajiro is a name unknown to English readers, but is one which stands out in contemporary history as that of the man who transformed Japan. "A military engineer, a bold traveller (at least, in wish), a poet, a patriot, a schoolmaster, a friend to learning, a martyr to reform, there are not many men dying at 70 who have served their country in such various characters. It is hard to say which is most remarkable—his capacity for command, which subdued his very jailers; his hot, unflagging zeal; or his stubborn superiority to defeat. He failed in each particular enterprise that he attempted, and yet we have only to look at his country to see how complete has been his general success." When we see all around us these brisk, intelligent students, with their strange, foreign air, we should never forget the hardships endured by Yoshida to gain for his native land the benefits that she now enjoys so largely. There is a quaint Japanese couplet quoted, spoken through the prison walls by Yoshida to a fellow-reformer on his way to the place of execution, which illustrates aptly the spirit of these heroes who sacrificed their lives for the reformation of their country—

"It is better to be a crystal and be broken
Than to remain perfect like a tile upon the housetop."

* "Men and Books." By R. Louis Stevenson. (Publishers: Chatto and Windus. 1 vol. 2s.)

### For Reading to the Sick.

THE PERFECT GIFT.

O YE, who taste that love is sweet,
Set way-marks for all doubtful feet
That stumble on in search of it.

Sing notes of love: that some who hear
Far off, inert, may lend an ear,
Rise up and wonder and draw near.

Lead life of love: that others who
Behold your life, may kindle too
With love, and cast their lot with you.

*Christina Rossetti.*

All through life there are wayside inns where man may
refresh his soul with love;
Even the lowest may quench his thirst at rivulets fed by
springs from above.

*Longfellow.*

The first note of the Spiritual Life, the first fruit of the Spirit, the outcome, that is, of the true and proper development of our life, will be—Love.

Let us try now and see one or two characteristics of Love' one or two signs of its indwelling, abiding presence. First of all Love will be thoughtful. "If God so loved us, we ought also to love one another." How much thoughtfulness may we trace in the Love of God! "God *so* loved us." There is all the thoughtfulness which lies around our creation, the beauty of the world we live in, the wonderful adaptation of our life, the daily tenderness and forethought of God, Who clothes the lily, Who feeds the ravens, and marks the fall of the sparrow to the ground, Who bids us cast out our cares and lay aside anxiety, for He is caring for us, and marking all our needs and wants.

Ah! Love throws open wide all those points of contact with our friend and our neighbour, that is with the world; and does it not need Love? "Nothing but the infinite pity is sufficient for the infinite pathos of human life." And the Spirit pours into the great machinery of our being, which finds it only too easy to be rough and hard, the germ of that "infinite pity" in His gift of Love. And equally true is it if we look towards our fellow-men, that Love is a foundation virtue. Ah! as we have looked into the machinery of our life we have found how much of it "has no meaning standing apart!" We see all the apparatus for the formation of friendship; the places where the tender bands of home have bound themselves in; the points of contact with our fellows which explain the existence of societies, of cities, and nations.—*Newbolt.*

The while my heart is decked and drest
For many a lief and welcome guest,
I would not have it over-filled;
Lest One who forth to-night doth fare,
And e'en with me to lodge hath willed,
Should pass, and shelter otherwhere.

*F. Langbridge.*

### Notes and Queries.

**The Editor is always willing to answer in this column, without any fee, all reasonable questions, as soon as possible.**
**But the following rules must be carefully observed:—**
**1. Every communication must be accompanied by the name and address of the writer.**
**2. The question must always bear upon nursing, directly or indirectly.**
**If an answer is required by letter a fee of half-a-crown must be enclosed with the note containing the inquiry, and we cannot undertake to forward letters addressed to correspondents making inquiries. It is therefore requested that our readers will not enclose either a stamp or a stamped envelope.**

*Vaccination.*

(187) I and my wife are in charge of a small-pox hospital and expecting cases of small-pox coming in at any moment. I wish to ask your opinion with regard to my wife being re-vaccinated. She is a staunch beliver in vaccination, but has not been well for some time and is at present four months advanced in pregnancy. 1. Would it be wise under such conditions to be re-vaccinated? 2. Would it have any detrimental effect on the fœtus? I may mention that she was re-vaccinated thirteen years ago.—*Inquirer.*

Your wife should, if possible, leave the hospital at once.

*Chilblains.*

(188) Can you give me a cure for chilblains? A dear little girl in the house where I am nursing is suffering very much from them, not broken; toes and heels very much inflamed. She is a very nervous child and her sore feet seem to take away all her confidence. —*Nurse Marie Rose.*

Any serious case of chilblain deserves medical treatment. We may state, however, that the general principles of treatment are in the first stage of the disease to restore the circulation by gentle friction, for which purpose some soothing liniment, such as the soap liniment with laudanum, may be employed. Later, a stimulating application, such as turpentine liniment, may be used, or the part may be painted with tincture of iodine, the parts being covered up in cotton wool or encased in plaster spread on wash-leather. When the chilblain becomes broken Friar's Balsam or resin ointment are useful applications. The great thing, however, is to prevent the occurrence of chilblains by keeping the extremities warm by wearing thick-soled boots, wash-leather socks, and lined gloves, and above all things by taking plenty of outdoor exercise.

*Starting a Home.*

(189) Can you tell me what qualifications are necessary in order to start a nurses' home and to enable one to give a certificate of proficiency to nurses? Would the house have to be registered? —*Mary.*

No private home can be a training school for nurses. You may take a house and engage a staff of nurses already trained without any formalities. Further, you may make private arrangements with a recognised training school to train and certificate your probationers, but any certificate you might give yourself would be valueless.

*Gout.*

(190) Is the Devonshire Hospital and Buxton Bath Charity free to sufferers from gout? Are there any other free charities for this class of disease?—*H. M. M.*

This institution is free for three weeks to patients with subscriber's recommendation. The Royal Mineral Water Hospital, Bath, is free to the poor of the United Kingdom.

*Perquisite.*

(191) 1. Will you kindly tell me if a nurse has the privilege of taking away the articles of clothing which she has removed from a dead body in preparing it for burial? I was under the impression that she was, and did so; now the article has been claimed by a detective, and I am anxious lest my reputation should suffer. 2. Could I call upon my employer and explain that I understood that the article in question was my perquisite?—*M. J. T.*

1. A nurse can only claim her fee for her services. 2. Yes.

**Standard Books of Reference.**

"The Nursing Profession: How and Where to Train." 2s. net; post free 2s. 4d.
"Burdett's Official Nursing Directory." 3s. net; post free, 3s. 4d.
"Burdett's Hospitals and Charities." 5s.
"The Nurses' Dictionary of Medical Terms." 2s.
"Burdett's Series of Nursing Text-Books." 1s. each.
"A Handbook for Nurses." (Illustrated). 5s.
"Nursing: Its Theory and Practice." New Edition. 3s. 6d.
"Helps in Sickness and to Health." Fifteenth Thousand. 5s.
"The Physiological Feeding of Infants." 1s.
"The Physiological Nursery Chart." 1s.; post free, 1s. 3d.
"Hospital Expenditure: The Commissariat." 2s. 6d.

All these are published by the SCIENTIFIC PRESS, Ltd., and may be obtained through any bookseller or direct from the publishers 28 and 29 Southampton Street, London, W.C.

## Figures and Tables

**Fig. 24. f1:**
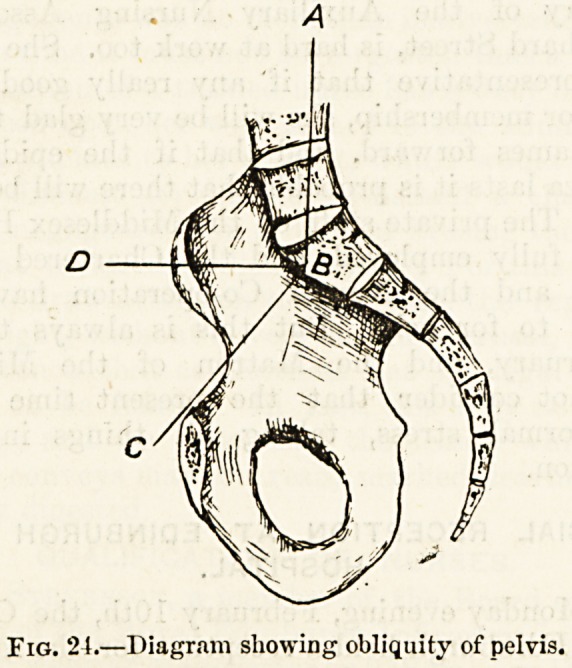


**Fig. 25. f2:**
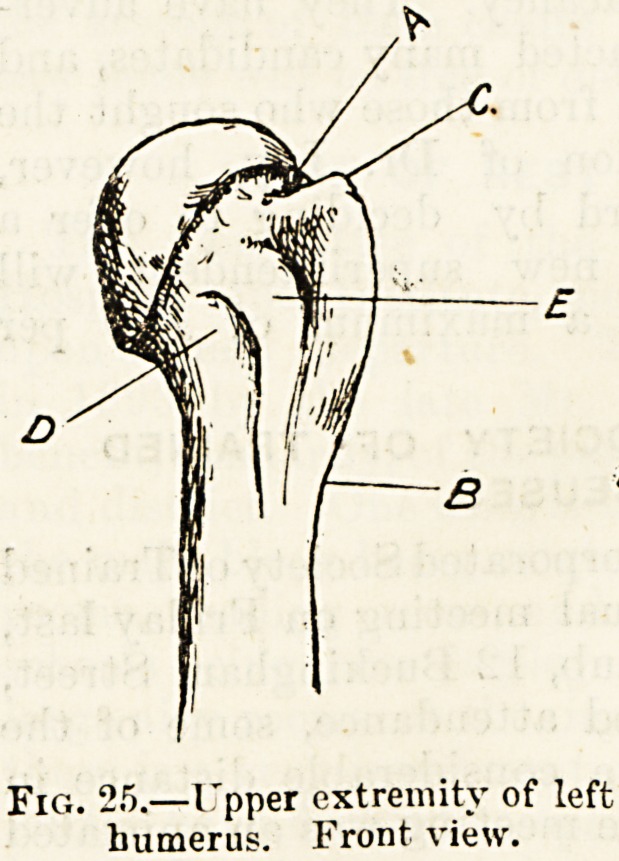


**Fig. 26. f3:**
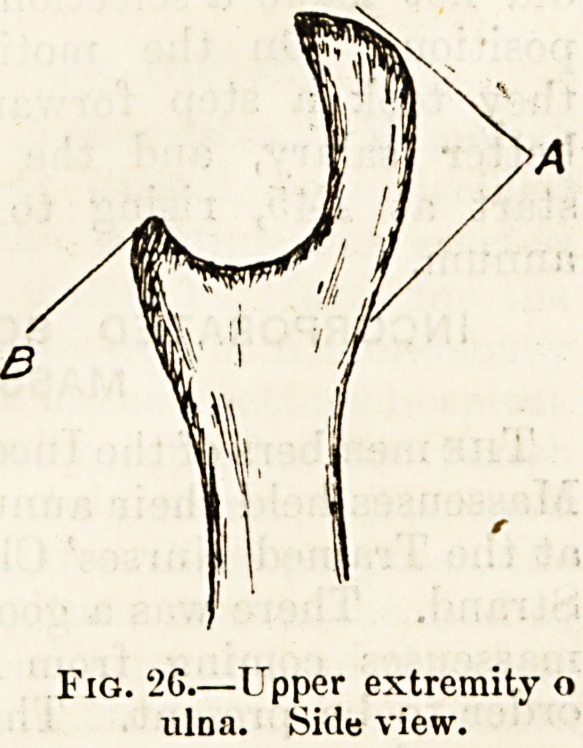


**Fig. 27. f4:**